# Bile acid-mediated gut-liver axis crosstalk: the role of nuclear receptor signaling in dynamic regulation of inflammatory networks

**DOI:** 10.3389/fimmu.2025.1595486

**Published:** 2025-05-19

**Authors:** Wenlong Yan, Kun Zhang, Jing Guo, Lingfen Xu

**Affiliations:** ^1^ Department of Pediatrics, Shengjing Hospital Affiliated to China Medical University, Shenyang, China; ^2^ The Center for Pediatric Liver Diseases, Children’s Hospital of Fudan University, Shanghai, China

**Keywords:** bile acid metabolism, gut-liver axis, FXR, TGR5, inflammatory network, intestinal flora, nuclear receptor signaling

## Abstract

Bile acids (BAs) are critical mediators of metabolic and immune regulation, influencing both liver and intestinal function. Their homeostasis, maintained through the enterohepatic circulation, is pivotal for immune-metabolic balance. BAs activate key receptors, including Farnesoid X Receptor (FXR) and TGR5, to modulate inflammation. FXR exerts anti-inflammatory effects by suppressing NF-κB signaling and cytokine production, whereas TGR5 primarily regulates NLRP3 inflammasome activation. Dysregulated BA signaling, driven by microbial dysbiosis, exacerbates inflammatory diseases like non-alcoholic fatty liver disease (NAFLD) and inflammatory bowel disease (IBD). This review explores the intricate roles of BAs in inflammation, highlighting the microbiome’s influence on BA metabolism and immune responses. Understanding the BA-immune axis offers new therapeutic avenues for modulating inflammation and improving clinical outcomes in inflammatory diseases.

## 1 Introduction

Bile acids (BAs) have emerged as central orchestrators of inflammation at the intersection of hepatic metabolism and mucosal immunity. Beyond their classical roles in lipid digestion, BAs function as pleiotropic signaling molecules through nuclear receptors and membrane-bound sensors, dynamically regulating inflammatory cascades across the gut-liver axis (1–3). The FXR-NF-κB axis exerts potent anti-inflammatory effects by suppressing pro-inflammatory cytokines and stabilizing epithelial junctions, while TGR5 activation antagonizes NLRP3 inflammasome assembly, a dichotomy that underscores BA’s immunomodulatory duality (4, 5).

Disruption of this delicate balance triggers pathological inflammation through multiple mechanisms: Impaired FXR signaling permits uncontrolled NF-κB activation (6), while TGR5 deficiency exacerbates hepatic inflammation via M1 macrophage polarization and caspase-1-dependent IL-1β maturation (7, 8). Emerging research reveals how microbial BA modifications dictate inflammatory outcomes: *Bacteroidetes*-mediated 7α-dehydroxylation generates pro-inflammatory DCA, whereas Lactobacillus-epimerized UDCA exhibits tissue-protective effects. This review synthesizes recent advances in BA-mediated immunomodulation, highlighting receptor-specific control of inflammatory pathways, microbiota-dependent BA transformation networks, and therapeutic opportunities targeting the BA-inflammatory axis in hepatobiliary and intestinal diseases.

## 2 Bile acid signal transduction: from metabolic regulation to immune modulation

### 2.1 Bile acid synthesis and enterohepatic circulation

The biosynthesis of primary bile acids is facilitated by two distinct cholesterol-derived metabolic pathways, both of which are evolutionarily conserved with species-specific regulation. The typically neutral pathway, dominant in human hepatocytes, begins with the evolutionarily conserved transformation of cholesterol in the endoplasmic reticulum (2). This particularly critical first step involves cytochrome P450 decatalyzing the rate-limiting 7α-hydroxylation of cholesterol to form 7α-hydroxyl cholesterol, a metabolic commitment point that is heavily regulated by FXR-mediated negative feedback on transcriptional inhibition, resulting in intermediates that then undergo a series of modifications (1, 9).

In the whole BA synthesis, the replacement acid pathway accounts for less than 10%, and this pathway mainly plays a role in the extrahepatic tissue. It relies on CYP27A1-mediated 27-hydroxylated cholesterol to form 27-hydroxylcholesterol. After that, this oxysterol is transported to the liver, where, CYP7B1 catalyzes stereospecific 7α -hydroxylation to produce 7α, 27-dihydroxycholesterol. Then, after side chain oxidation and peroxisome processing, the final product is CDCA. When the classical pathway is inhibited, this acid replacement pathway acts as a compensatory mechanism during cholestasis. This metabolic plasticity of the microorganisms produces a diverse pool of bile acids with different physical and chemical properties (10). Such as critical micelle concentration and hydrophobicity index, and their receptor affinity is different, such as selectivity for FXR and selectivity for TGR5, so that the host metabolism can be more subtle regulation (1, 2, 11).

### 2.2 Bile acids activate multiple receptors to regulate metabolism and inflammatory changes

#### 2.2.1 FXR

The Farnesoid X receptor (FXR) serves as the central regulatory node for bile acid sensing, exhibiting ligand-dependent activation kinetics with distinct activation thresholds for different bile acids such as cholic acid. This molecular recognition event initiates a transcriptional cascade characterized by the transactivation of the small heterodimer partner (SHP) and the competitive displacement of HNF4α from the CYP7A1 promoter, thereby establishing an autoregulatory loop for cholic acid homeostasis. Notably, intestinal FXR activation induces the secretion of fibroblast growth factor 19 (FGF15 in mice), which subsequently activates hepatic FGFR4/β-Klotho receptor complexes via portal circulation. This enterohepatic signaling axis mediates CYP7A1 inhibition through an ERK1/2 phosphorylation cascade, demonstrating a critical cross-talk between intestinal and hepatic compartments (12).

FXR exerts pleiotropic anti-inflammatory effects through multilayered regulatory mechanisms: (1) Direct suppression of NF-κB signaling via p65 subunit sequestration coupled with proteasomal degradation pathways; and (2) Transcriptional repression of pro-inflammatory cytokines (TNF-α, IL-6, IL-1β) through chromatin-bound SHP complexes (13). Furthermore, FXR coordinates hepatic detoxification processes by enhancing bile salt export pump (BSEP)-mediated biliary excretion while inhibiting basolateral uptake via the sodium taurocholate cotransporting polypeptide (NTCP), thereby preventing intracellular bile acid overload (14). NTCP serves as a multifunctional molecule with dual physiological roles: as the primary mediator of sodium-dependent bile acid uptake (15) and as the cellular entry receptor for hepatitis B virus through its interaction with the myristoylated preS1 domain. This vulnerability has been exploited therapeutically with agents like Myrcludex B, which simultaneously blocks viral entry while modulating bile acid-induced interferon responses (16, 17).

Intestinal FXR contributes to epithelial barrier integrity through two principal mechanisms: stabilization of tight junction proteins (claudin-1/occludin complexes) and angiopoietin-mediated antimicrobial peptide secretion. Additionally, FXR modulates gut microbial bile acid metabolism by regulating substrate availability (18). Emerging research has identified novel regulatory axes in FXR biology, including the discovery of liver-specific enhancer RNAs such as Fincor. This long non-coding RNA is induced by the FXR agonist tofexiflex, and CRISPR/Cas9-mediated ablation studies have established its essential role in NASH resolution. Genetic deletion of Fincor completely abrogates the therapeutic effects of tofexiflex, including: (1) attenuation of hepatic steatosis via SREBP1c downregulation; (2) inhibition of fibrogenesis through TGF-β pathway suppression; and (3) resolution of lobular inflammation via CCL2/MCP-1 inhibition (19).

#### 2.2.2 TGR5 in inflammatory and immunomodulatory processes

TGR5 is a key bile acid receptor, which plays pivotal roles in inflammatory and immunomodulatory processes through metabolic and microbiota regulation. TGR5 downregulation in both non-alcoholic steatohepatitis (NASH) patients and murine models TGR5-knockout (TGR5^−/−^) mice exhibited exacerbated hepatic injury, elevated proinflammatory cytokines, and enhanced M1 macrophage polarization. Mechanistically, TGR5 deficiency promoted NLRP3 inflammasome activation and caspase-1 cleavage, intensifying M1 polarization. Methionine-choline deficient (MCD) diet-induced NASH models revealed more severe hepatic steatosis and inflammation in TGR5^−/−^ mice compared to wild-type (WT) controls, with significantly higher NAFLD activity scores. Quantitative analysis showed TGR5 deletion increased hepatic levels of TNF-α and IL-6 while reducing IL-4 and IL-10 (20). Notably, TGR5^−/−^ livers displayed enhanced macrophage infiltration and M1 polarization. Further investigations identified TGR5-mediated suppression of NLRP3 inflammasome activation as the regulatory mechanism. Both TGR5^−/−^ mice and bone marrow-derived macrophages (BMDMs) exhibited upregulated NLRP3, caspase-1, IL-1β, and IL-18 expression under MCD diet or palmitic acid (PA) stimulation pharmacological inhibition of NLRP3 using CY-09 effectively suppressed inflammatory mediators and promoted M2 polarization. Clinical correlations in NASH patients confirmed inverse relationships between TGR5 expression and NLRP3 activation/M1 polarization (3, 20–22).

By studying the endoplasmic reticulum (ER)-mitochondrial coupling mediated by grp75, the protective mechanism of TGR5 in diabetic retinopathy. After TGR5 is activated, it will destroy the IP3R1-GRP75-VDAC1 axis, thus weakening the situation of diabetic retinopathy. It can also reduce the Ca ^2 +^ flux from the endoplasmic reticulum to the mitochondria, and the subsequent problem of mitochondrial Ca ^2 +^ overload. This regulatory effect can prevent the opening of mitochondrial permeability transition pores, prevent the release of mitochondrial DNA, and inhibit CGAS-STING-mediated inflammation. TGR5 agonists can improve retinal damage in diabetic models, while STING inhibitors can improve retinal damage in diabetic models (23). Microbial deoxycholic acid can alleviate mastitis induced by Staphylococcus aureus through the TGR5-cAMP-PKA-NF-κB/NLRP3 signaling pathway (21, 24). Ursodeoxycholic acid can reduce mastitis induced by *Staphylococcus aureus* by activating TGR5-NF-κB. And restore short-chain fatty acids to reduce neonatal calf colitis induced by enteric aggregative Escherichia coli that produces ultra-broad spectrum beta-lactamase (25). Intestinal microbiota - bile acid - fxr/TGR5 axis can improve intestinal barrier function, and can also inhibit colon inflammation (26).

#### 2.2.3 Others critical mediators

Activated T cell family nuclear factors have become key mediators in bile acid-induced inflammatory processes. Studies on the mechanism have shown that human and mouse hepatocytes exposed to bile acid concentrations related to pathological simulation can induce nuclear translocation of NFATC3. It is also accompanied by upregulation of IL-8, CXCL2 and CXCL10 chemokine networks (27). Pharmacological NFAT inhibition or NFATC3 genetic ablation substantially attenuates this inflammatory cascade, establishing causal linkage. It has been clinically confirmed that nuclear NFATC3 accumulation exists in PBC/PSC liver specimens. It showed a strong positive correlation with IL-8 expression gradient. Molecular validation by luciferase reporter gene assay and CHIP-seq confirmation demonstrated that bile acids are directly involved in the NFAT response element within the IL-8 promoter, and site-specific mutations of this cis-regulatory motif would eliminate bile acid-driven transcriptional activation, thus delineating the molecular logic of the mechanism (27, 28). This axis of inflammation mediated by fat is not only limited to hepatobiliary pathology, but also contributes to ZTHE development of colorectal cancer and the progression of viral hepatitis through conserved regulatory topology (29, 30).

The expression of intestinal antimicrobial peptides has prominent spatial and temporal heterogeneity, and its regulatory structure has not yet been determined (31). Cutting-edge single-cell transcriptomics uncovers bile acid transcription factors as chromatin topological determinants of AMP heterogeneity, which is different from the typical signaling paradigm (32). BATF regulates amp by means of atypical, bile acid-independent mechanisms related to lineage commitment decisions rather than direct receptor activation. Single-cell ATAC-seq analysis also found that the chromatin accessibility landscape predetermines the regulatory potential of BATF through epigenetic prepatterns, and that this double-layer regulatory pattern, that is, the combination of chromatin topology and BATF activity, controls AMP zoning after birth and coordinates the ontogenetic process of fetal intestinal immunity (32). The discovery of bile acid-mediated epigenetic priming provides mechanistic insight into how microbial metabolites shape mucosal defense systems during developmental window.

### 3.1 Bile acids on the intestinal flora and liver function of cholestasis

In infants with intrahepatic cholestasis, such as in familial cholestasis syndromes, genetic defects in bile acid synthesis enzymes or transporters lead to the accumulation of cytotoxic bile acids (BAs) within hepatocytes and impaired biliary secretion. This metabolic disturbance triggers the overexpression of nuclear factor of activated T-cells (NFAT) in hepatic parenchymal cells, exacerbating liver inflammation (27, 33). Persistent inflammatory signaling may progress to fibrotic remodeling, further aggravating hyperbilirubinemia and cholestatic liver injury (34). Under normal physiological conditions, primary bile acids are efficiently metabolized into secondary bile acids by gut microbiota in the intestinal lumen (35). Secondary bile acids exert protective effects on the biliary tract through TGR5 which operate via two key pathways: one is the enhancement of epithelial barrier function through the upregulation of tight junction proteins, including binding adhesion molecule-a (JAM-A); another is the stimulation of biliary proliferation via reactive oxygen species (ROS)-dependent Src/EGFR activation (36, 37).

In addition, BA can activate FXR in the gut and play an antibacterial role by inhibiting *enterococcus* and *Clostridium difficile*, which has been mentioned in related studies (38, 39). Homeostasis of BA can regulate mucosal immunity in some ways, such as TH17/Treg cell (40) balance regulation and epithelial β-defensin production (41). And with TGR5-dependent NLRP3 inflammasome inhibition (20). However, stone cholic acid shows a relatively good anti-inflammatory effect, and when there is a cholestatic BA imbalance, it will make intestinal inflammation more serious, and also reduce the number of symbiotic bacteria such as *Bifidobacterium adolescentis*, *Lactobacillus plantarum*, and *Faecalibacterium prausnitzii (* 26, 42).

### 3.2 The impact of the intestinal microbiota on BA and liver function in cholestasis

#### 3.2.1 Microbial-BA interactions in clinical cholestasis

The changes in the gut microbiota during cholestasis also affect BA The gut microbiota serves as a central regulator of bile acid (BA) homeostasis through enzymatic activity within the intestinal lumen. Wang et al. (43) systematically investigated microbial-BA interactions in infants with cholestatic jaundice (CJ) via integrated 16S rRNA sequencing and fecal BA profiling. Their analysis revealed significant reductions in primary and secondary BA levels, accompanied by a metabolic shift from classical to alternative BA synthesis pathways. Notably, CJ infants exhibited microbial communities enriched with *Clostridium* and *Streptococcus* alongside depleted *Bifidobacterium*, with these dysbiotic patterns strongly correlating to disrupted BA profiles and elevated hepatic dysfunction markers. Liu et al. (44) further dissected microbiome differences across biliary atresia, non- biliary atresia cholestasis (IC), and healthy cohorts using 16S rDNA sequencing. The IC group displayed a distinct microbial architecture dominated by *Enterobacteriaceae*, showing positive associations with serum transaminases and total bilirubin. These findings underscore divergent enterohepatic BA cycling mechanisms between obstructive and metabolic cholestasis. In addition, the changes of bile acid metabolism mediated by *Lactobacillus* and *clostridium* are also an important part of the treatment of IBD (26).

#### 3.2.2 Enzymatic regulation of BA pools

The gut microbiota exerts enzymatic control over bile acid pool composition through three phylogenetically conserved biotransformation cascades. First, bile salt hydrolase (BSH) produced by commensal *Clostridium* and Bacteroides catalyzes the hydrolytic deconjugation of primary BAs, cleaving glycine/taurine moieties from steroid cores to generate unconjugated species with enhanced membrane permeability and signaling potential (45). Second, hydroxysteroid dehydrogenase (HSDH) derived from *Lactobacillus* mediates stereoselective 7β-epimerization via NADPH-dependent redox cycling, converting CDCA into its 7β-hydroxy isoform DCA - a secondary BA with increased hydrophobicity and receptor-binding selectivity (46). Third, The specialized anaerobic 7α-dehydroxylation of Bacteroides, which irreversibly removes the C7 hydroxyl group from cholic acid, relies on sequential ketoenol tautomerism and reduction elimination steps to produce cholic acid, resulting in the most hydrophobic and cytotoxic BA species, which has been documented in relevant studies (9, 11). These three microbial enzymes work together to determine the structural diversity, physical and chemical properties and endocrine functions of the enterohepatic BA pools.

#### 3.2.3 Dysbiosis-exacerbated enterohepatic pathology

*Bifidobacterium* depletion potentiates FXR signaling through diminished tauro-β-muricholate and elevated FGF15 production (47), thereby suppressing primary bile acid synthesis while promoting the dominance of alternative acidic biosynthetic pathways - ultimately reducing CA/CDCA ratios (43). Under cholestatic conditions, *Clostridium/Enterococcus/Clostridioides difficile* expansion elevates DCA levels that drive TLR2-mediated macrophage polarization, exacerbating intestinal inflammation and microbiota dysbiosis (40, 48). The BAIA1 gene encoded by *Firmicutes* critically mediates the enzymatic reduction of 3-oxo-allo-deoxycholic acid (3-oxo-allo-DCA) to allo-deoxycholic acid (allo-DCA), and similarly converts 3-oxo-allo-lithocholic acid to allo-LCA. Besides, BAIA1 overexpression demonstrates a significant association with colorectal carcinogenesis (29, 49).

#### 3.2.4 Gut-liver axis in cholestatic progression

**Figure 1:**
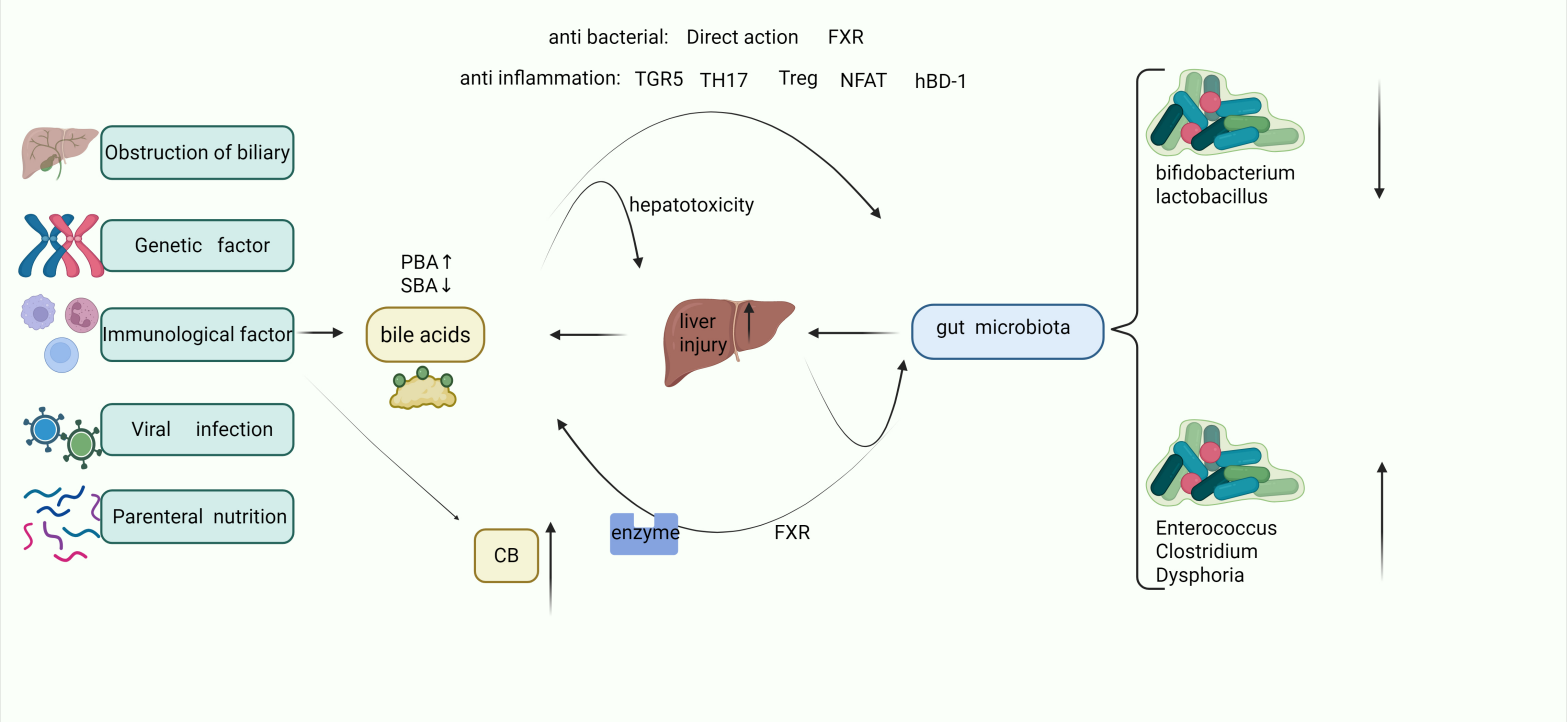
Bile acid-mediated gut-liver axis crosstalk.

The hepatoprotective capacity of commensal microbiota is evidenced in bile duct ligation (BDL) models: Germ-free mice exhibit exacerbated cholestatic injury compared to conventionalized counterparts, with microbial colonization attenuating bile infarct progression through coordinated activation of hepatic regenerative programs, maintenance of inflammatory homeostasis, lipidomic reprogramming, and optimization of mitochondrial respiratory efficiency (50). Concurrent Kupfer cell activation propagates hepatobiliary injury feedforward through bilirubine-induced inflammatory cascade initiation, cytokine storm cascade disruption of the intestinal barrier, and secondary cholic acid metabolism disorders (7, 51).Pathological microbial shifts facilitate endotoxin translocation across compromised intestinal epithelium, establishing a self-perpetuating gut-liver injury axis that perpetuates cholestasis and hyperbilirubinemia (52, 53) (**Figure 1**).

## 4 Current challenges in gut-liver axis research and development pathways

Bile acid metabolites are considered promising biomarkers. Relevant studies found different bile acid characteristics, which can be used to predict anti-TNF treatment response in Crohn’s disease. Serum deoxycholic acid levels were elevated in those who did respond to treatment compared to those who did not respond to treatment (54). It should be noted that patients with fecal cholic acid concentrations below 0.05μM had an increased likelihood of developing treatment resistance. Combining specific bile acid derivatives to form a biomarker panel was more predictive of treatment outcome. These findings suggest that there is metabolic cross-talk between bile acid synthesis in the liver and microbial modification in the gut. Although some progress has been made in bile acid research, bile acid research still faces key challenges, such as the effects of high-fat diets, which temporarily alter bile acid profiles depending on mechanisms involving intestinal permeability and microbial gene expression. In MASH (55) and liver fibrosis (56), obecholic acid (OCA) can activate FXR and then inhibit CYP7A1, reduce BAs synthesis, reduce the toxicity of bile acid accumulation in hepatocytes, down-regulate pro-inflammatory factors, such as alleviating liver inflammation. OCA can also cause side effects such as pruritus, dyslipidemia, gastrointestinal discomfort and abnormal liver function.

Recent therapeutic development strategies have focused on multimodal interventions. An engineered probiotic expressing 7α-dehydroxylase has been shown to regulate intestinal bile acid composition by increasing secondary bile acid production, and fecal microbiota transplantation in combination with FXR agonists has a synergistic effect in reducing portal endotoxin (57, 58). Advances in mass spectrometry imaging techniques that enable high-resolution spatial mapping of bile acid distribution in intestinal crypt villi structures complement deep learning approaches that integrate metabolomic data for personalized treatment optimization, and these innovations represent the convergence of microbiology, bioengineering, and computational biology in advancing precision medicine (55, 59).

## 5 Conclusion

Bile acids (BAs) play a dual role in immune-metabolic regulation, acting as both signaling molecules and modulators of gut-liver crosstalk. Through nuclear receptors and membrane-bound receptors, BAs regulate inflammation, barrier integrity, and microbial homeostasis. Dysregulated BA signaling drives cholestasis, NAFLD, and IBD by disrupting FXR-mediated anti-inflammatory pathways and promoting NLRP3 inflammasome activation via TGR5 suppression. The gut microbiota critically shapes BA pools through enzymatic modifications, linking dysbiosis to disease progression. Emerging therapies, including FXR agonists, engineered probiotics, and microbiota-targeted interventions, offer promise but face challenges such as side effects and interpatient variability. Future research should integrate multi-omics, spatial metabolomics, and AI-driven modeling to optimize precision medicine approaches for BA-related disorders.
